# Small molecule inhibitor of tau self-association in a mouse model of tauopathy: A preventive study in P301L tau JNPL3 mice

**DOI:** 10.1371/journal.pone.0286523

**Published:** 2023-08-09

**Authors:** Eliot J. Davidowitz, Patricia Lopez, Heidy Jimenez, Leslie Adrien, Peter Davies, James G. Moe

**Affiliations:** 1 Oligomerix, Inc., White Plains, NY, United States of America; 2 Oligomerix, Inc., Bronx, NY, United States of America; 3 The Litwin-Zucker Research Center for the Study of Alzheimer’s Disease, The Feinstein Institutes for Medical Research, Northwell Health, Manhasset, NY, United States of America; McGill University, CANADA

## Abstract

Advances in tau biology and the difficulties of amyloid-directed immunotherapeutics have heightened interest in tau as a target for small molecule drug discovery for neurodegenerative diseases. Here, we evaluated OLX-07010, a small molecule inhibitor of tau self-association, for the prevention of tau aggregation. The primary endpoint of the study was statistically significant reduction of insoluble tau aggregates in treated JNPL3 mice compared with Vehicle-control mice. Secondary endpoints were dose-dependent reduction of insoluble tau aggregates, reduction of phosphorylated tau, and reduction of soluble tau. This study was performed in JNPL3 mice, which are representative of inherited forms of 4-repeat tauopathies with the P301L tau mutation (e.g., progressive supranuclear palsy and frontotemporal dementia). The P301L mutation makes tau prone to aggregation; therefore, JNPL3 mice present a more challenging target than mouse models of human tau without mutations. JNPL3 mice were treated from 3 to 7 months of age with Vehicle, 30 mg/kg compound dose, or 40 mg/kg compound dose. Biochemical methods were used to evaluate self-associated tau, insoluble tau aggregates, total tau, and phosphorylated tau in the hindbrain, cortex, and hippocampus. The Vehicle group had higher levels of insoluble tau in the hindbrain than the Baseline group; treatment with 40 mg/kg compound dose prevented this increase. In the cortex, the levels of insoluble tau were similar in the Baseline and Vehicle groups, indicating that the pathological phenotype of these mice was beginning to emerge at the study endpoint and that there was a delay in the development of the phenotype of the model as originally characterized. No drug-related adverse effects were observed during the 4-month treatment period.

## Introduction

There is an unmet and critical need for a disease-modifying therapy for Alzheimer’s disease (AD) and related dementias. AD is the only one of the 10 leading causes of death in the United States that cannot be prevented, slowed, or cured [1]. Long-term treatment for chronic diseases such as AD requires therapies that are safe, effective, and economically feasible—especially for early preventive treatment strategies. The amyloid cascade hypothesis has been the dominant paradigm for drug discovery for AD, but recent advances in the understanding of tau biology in neurodegenerative diseases and difficulties of amyloid-directed immunotherapeutics have heightened interest in tau as a target for drug discovery [2–7], particularly tau oligomers [8, 9].

Microtubule-associated protein tau is localized primarily in the axons of neurons, where it is thought to modulate microtubule stability [10] or the dynamics of labile microtubule ends [11] and may have a role for enabling signaling pathways [12]. Tau has been implicated in the pathogenesis of multiple neurodegenerative diseases associated with accumulation of abnormal tau species; these diseases are collectively called *tauopathies*. Mutations in microtubule-associated protein tau can cause altered ratios of 3- or 4-repeat isoforms of tau protein or changes in the structure of tau, both of which can cause tau to aggregate and cause a range of rare inherited tauopathies. In AD, tau pathology is driven by numerous posttranslational modifications that lead to loss of normal function, gain of toxic function, or both [13]. Neurofibrillary tangles, composed primarily of tau, accumulate in a highly reproducible spatiotemporal pattern starting in the transentorhinal/entorhinal regions and spread through the hippocampal structure to the neocortex [14], demonstrating a close association between tau aggregation and AD progression. Tau prion-like propagation through neuronal communication pathways uses a seeding mechanism of templated misfolding [15–17] and is accelerated by the presence of brain beta-amyloid [18], suggesting that this pathway may be a viable target for the development of disease-modifying therapies for AD. Furthermore, data from aging humans show that tau phosphorylation occurs in the transentorhinal/entorhinal regions a decade before the appearance of amyloid plaques, indicating that tau pathology may initiate late onset of AD [19].

Multiple studies have shown that tau oligomers are most closely correlated with neuronal loss and memory impairment [20–25]. A recent review described the neurotoxic effects of tau oligomers on the genome, mitochondria, cell signaling, synaptic plasticity, microtubule assembly, cytoskeleton, and protein-clearance mechanisms in detail [26]. We have shown that tau oligomers disrupt neuronal signaling and inhibit the formation of memory in mice [27, 28]. Memory formation was impaired after administration of oligomeric tau to hippocampi, areas of the brain involved in short-term memory formation. However, similar treatment with tau monomer (ie, tau that did not self-associate) had no effect. This impairment of memory was also found using oligomers formed from hyperphosphorylated tau purified from human AD brain specimens. Memory-specific mechanisms involved in gene regulation were shown to be disrupted by these extracellular tau oligomers. Our group has found that certain forms of tau oligomers are toxic when applied to cultured neurons, whereas tau monomer was not toxic at the same concentrations [29]. The kinetics of tau binding/unbinding to microtubules is rapid, but tau self-association is the rate-limiting step in its aggregation and may be a suitable target for an inhibitor.

In the present study, a small molecule approach was used to prevent tau self-association, which is understood to be the initiating event in the formation of tau oligomers and larger tau aggregates. OLX-07010 is an orally bioavailable, small molecule drug that inhibits tau self-association to prevent the initial step of tau aggregation and subsequently reduces the accumulation of all tau aggregates, which appear to be responsible for neuronal damage, spread of tau pathology, and impaired cognitive function. In a previous study, OLX-07010 was shown to prevent the accumulation of self-associated tau and detergent insoluble tau aggregates in human tau (htau) mice [30]. htau mice express both 3- and 4-repeat tau isoforms without mutations and in the absence of endogenous murine tau, and represent tau aggregation in AD. However, due to species-specific differences in splicing between humans and mice, the 3-repeat tau isoforms are relatively more abundant in the htau model [20]. Additionally, male htau mice have been observed to develop more tau pathology than female mice [30, 31], and the accumulation of tau pathology is relatively slow in htau mice compared with other mouse models of tauopathy, with tau mutations facilitating its aggregation. To address these issues, we performed studies using JNPL3 mice that exogenously express the 4R0N human tau construct with the P301L mutation that causes tau to aggregate at a faster rate. Homozygous female JNPL3 mice were chosen for the study because tau pathology is more pronounced in these mice [32, 33], making it a more challenging target for our small molecule inhibitor. Original characterization of the tau P301L JNPL3 mouse model [34] showed deposition of neurofibrillary tangles in the spinal cord, hindbrain, and diencephalon, as well as accumulation of granular aggregates in the cortex, hippocampus, and basal ganglia. Hind limb motor dysfunction corresponded to the loss of motor neurons in the spinal cord [34], and gait initiation failure was associated with hyperphosphorylated paired helical filaments in the brainstem of JNPL3 mice [35]. Although motor dysfunction in homozygous female JNPL3 mice may become apparent as early as 9 months of age, the underlying tau pathology was shown to develop by 7 months of age, which is the endpoint in this preventive study.

The purpose of this study was to evaluate OLX-07010, a small molecule inhibitor of tau self-association, for the prevention of tau aggregation in JNPL3 mice, representative of inherited forms of 4-repeat tauopathies with the P301L tau mutation, such as progressive supranuclear palsy and frontotemporal dementia.

## Materials and methods

### Animal model

Female homozygous tau P301L JNPL3 mice were purchased from Taconic Biosciences, and at 2 months of age were shipped to The Feinstein Institutes for Medical Research, where they were aged to 3 months prior to the start of treatment. Mice were examined, handled, and weighed before study initiation and were examined at least once a week during treatment to ensure adequate health and suitability. Rooms were maintained with 12-hr light/dark cycles, at a temperature of 20 to 23°C, with a relative humidity of approximately 50%. Food and water were provided in the home cage ad libitum for the duration of the study. All experiments were conducted in compliance with the Feinstein Institutes for Medical Research Institutional Animal Care and Use Committee.

### Study design

Female homozygous tau P301L JNPL3 mice were treated from 3 to 7 months of age; the compound was administered in the feed. The mice were separated into 4 groups: a Baseline group (n = 15, euthanized at 3 months of age), a Vehicle group (n = 20, euthanized at 7 months of age), and 2 Treatment groups (30 and 40 mg/kg doses, n = 20 per group, euthanized at 7 months of age). Mice were euthanized under deep anesthesia with isoflurane using cervical dislocation. The dose was estimated using an average body weight and an average daily consumption of feed. Giving the treatment in feed enabled stress- and harm-free administration compared with administering via oral gavage or intraperitoneal injection and was required for Institutional Animal Care and Use Committee approval for the protocol with 5 months of daily treatment. The study was performed independently at The Feinstein Institutes for Medical Research by Peter Davies, PhD. The batches of feed for the Vehicle and treatment groups were prepared by Research Diets, Inc., and were blinded by Oligomerix prior to delivery to the Davies laboratory. Manufacture of OLX-07010 batch AQ-007 was performed by Fox Chase Chemical Diversity Center (Doylestown, PA), and purity was confirmed to be 100% by high-performance liquid chromatography.

The primary endpoint of the study was statistically significant reduction of insoluble tau aggregates in the brains of the mice. The secondary endpoints were dose-dependent reduction of insoluble tau aggregates, reduction of phosphorylated tau (ptau) in the insoluble and heat stable fractions, and reduction of total tau. The biochemical data were unblinded after the analyses were completed.

#### Open standard diet (Product D11112201N) for vehicle feed

Manufacture of lots of feed was performed by Research Diets, Inc., (New Brunswick, NJ). 200 mg OLX-07010/kg Open Standard Diet (Product D16110202N) for estimated daily dose for mice in the 40 mg/kg dose group was based on average weight and daily consumption. 150 mg OLX-07010/kg Open Standard Diet (Product D19011402) for estimated daily dose for mice in the 30 mg/kg dose group was based on average weight and daily consumption. Sample feed pellets at each dose were randomly selected and assayed in duplicate to qualify the levels and stability of the compound in the feed by liquid chromatography with tandem mass spectrometry (LC/MS/MS) analyses of the feed samples (Quintara Discovery, Hayward, CA).

#### Enzyme-linked immunosorbent assays

Details of sample preparation and enzyme-linked immunosorbent assays (ELISAs) have been described in previous publications [30, 36]. Three types of brain preparations were performed for assays to determine the levels of total self-associated tau, Sarkosyl-insoluble tau aggregates, and soluble tau in the heat-stable (HS) fractions [36]. For self-associated tau, brain tissue from the forebrain or hippocampus was homogenized in 10 volumes of cold Tris-buffered saline, pH 7.4, containing protease and phosphatase inhibitors, cleared by low-speed centrifugation, and stored at -80°C. To prepare Sarkosyl-insoluble tau, the cleared homogenate was incubated for 10 min with 1% Sarkosyl. The insoluble tau was pelleted by ultracentrifugation at 200,000 x g for 30 min and washed with a homogenization buffer. The pellet was suspended in 1x Laemmli sample buffer and heated for 10 min at 100°C to dissociate the tau for analysis by ELISA. HS fractions of brain tissue were prepared by homogenization in 10 volumes of ice-cold buffer (50 mM Tris, pH 7.5, 0.8 M NaCl, 5% β-mercaptoethanol), and the cleared homogenate was heated at 100°C for 10 min to precipitate most proteins. Tau has the special characteristic of remaining soluble under these conditions, facilitating its purification in the supernatant/HS fraction. Supernatants were cooled and dialyzed against 50 mM Tris-buffered saline, pH 7.5, with 1 mM EDTA, 0.1 mM PMSF.

#### Antibodies

The tau antibodies used in these studies were all developed, produced, and formatted for assays in the laboratory of Peter Davies, PhD, Director, Litwin-Zucker Center for Alzheimer’s Disease & Memory Disorders, The Feinstein Institutes for Medical Research (Manhasset, NY). Pan-tau antibody monoclonal antibody (mAb) DA31 epitope spans amino acids 150–190 in 4R2N tau. Additional assays were performed for phospho-tau epitopes pertinent in AD using mAbs PHF-1 (pSer-396/404), CP13 (pSer-202), and RZ3 (pThr-231).

#### Tau ELISAs

Tau sandwich ELISAs for total tau and ptau epitopes were performed with capture antibodies DA31, PHF1, CP13, and RZ3; all ELISAs used reporter antibody DA9-HRP, as described. The tau mono-antibody ELISA was performed using DA9 for capture antibody and DA9-HRP for reporter antibody. The basis for mono-antibody ELISAs detecting self-associated protein is the use of the same mAb specific for a single epitope in the protein for both the capture and reporter antibodies. For rigor, the reproducibility of the ELISA formats used in these studies and their performance was evaluated by repeating the assays with control specimens from 7-month-old mice. There was no signal for any of the ELISA formats for total tau, ptau, or self-associated tau from the tau knockout mice, but there were higher levels of signal in the aged htau and JNPL3 mice (expressing the htau 4R0N isoform with the P301L mutation) over the wild-type mice, indicating the method was robust and reproducible [30]. Quantification of compound levels in mice sera was performed using LC/MS/MS at Quintara Discovery (Hayward, CA).

### Statistical analysis

All statistical analyses were performed using GraphPad Prism 9.3 (GraphPad Software, San Diego, CA). Outliers were removed using the ROUT (Q = 1%) method. Two-tailed unpaired *t* tests were used for comparisons of two groups with normal Gaussian distributions, while nonparametric analyses were performed with the Mann–Whitney *U* test. One-way analysis of variance (ANOVA) was used for parametric analyses and the Kruskal-Wallis test was used for nonparametric analyses. Error bars indicate standard deviation. Correlation analyses were performed with Spearman rank correlation (r). Overall trend of the data was demonstrated by a simple linear regression wherein the line represents the best fit and simple linear regression of the data were presented as mean ± standard error of the mean, with each dot representing an individual mouse. Comparisons were considered statistically significant at an α level of P < .05.

## Results

### Treatment caused no observable adverse events

**Fig 1:**
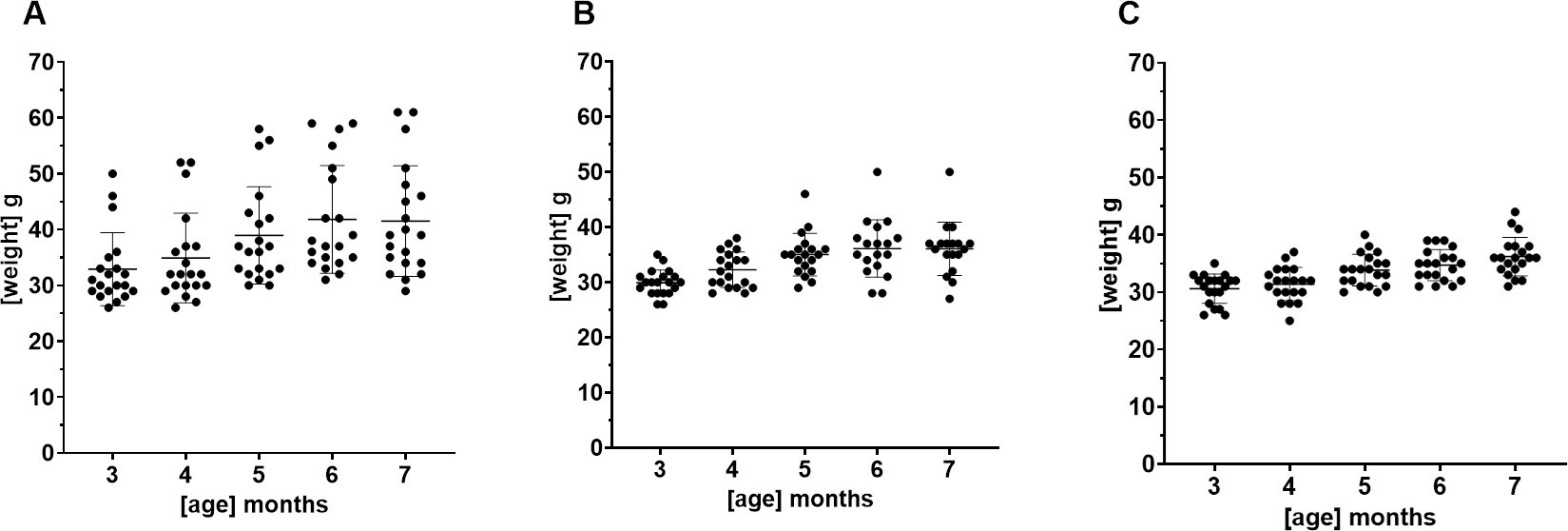
Mouse weights in the Vehicle and treatment groups during the treatment phase of the study. The diets for the Treatment groups were analyzed by LC/MS/MS to qualify the compound levels before the study and to determine compound level stability after the study. Diets for the (A) Vehicle group, (B) 30 mg/kg dose group, and (C) 40 mg/kg dose group were formulated to provide the correct dose based on average mouse weight and daily consumption. To determine the actual compound content in the diets, 2 pellets of feed were analyzed for each diet. Analysis of compound levels in the diet for the 30 mg compound/kg (mouse) dose showed that the compound concentration was 160 mg compound/kg diet prior to treatment, for an estimated dose of 32 mg/kg. After the study, the compound concentration was decreased to 114 mg compound/kg diet, for an estimated dose of 22.8 mg/kg. Analysis of compound levels in the diet for the 40 mg/kg dose had an initial concentration of 221 mg compound/kg diet, for an estimated dose of 44.2 mg/kg; after the study, the concentration was 160 mg compound/kg diet, for an estimated dose of 32 mg/kg.

No drug-related adverse effects were observed during the 4-month treatment period. Mice were randomly assigned to Baseline, Vehicle, or one of the 2 Treatment groups. Mouse weights were generally stable during treatment (Fig 1). Samples for biochemical analysis were obtained from 15 mice in the Baseline group, 20 mice in the Vehicle group, 18 mice in the 30 mg/kg dose group, and 20 mice from the 40 mg/kg dose group. There was attrition of 2 mice in the 30 mg/kg dose group; this was unrelated to treatment.

### Treatment prevented accumulation of Sarkosyl-insoluble tau in the hindbrain

We hypothesized that treatment with an inhibitor of tau self-association would affect the entire aggregation process, leading to the reduction of self-associated tau and the increasingly larger aggregates that accumulate in AD and related tauopathies. An accepted standard for evaluating treatments targeting tau aggregation is the reduction of Sarkosyl-insoluble aggregates of tau, which represent the end products of the tau aggregation cascade.

**Fig 2:**
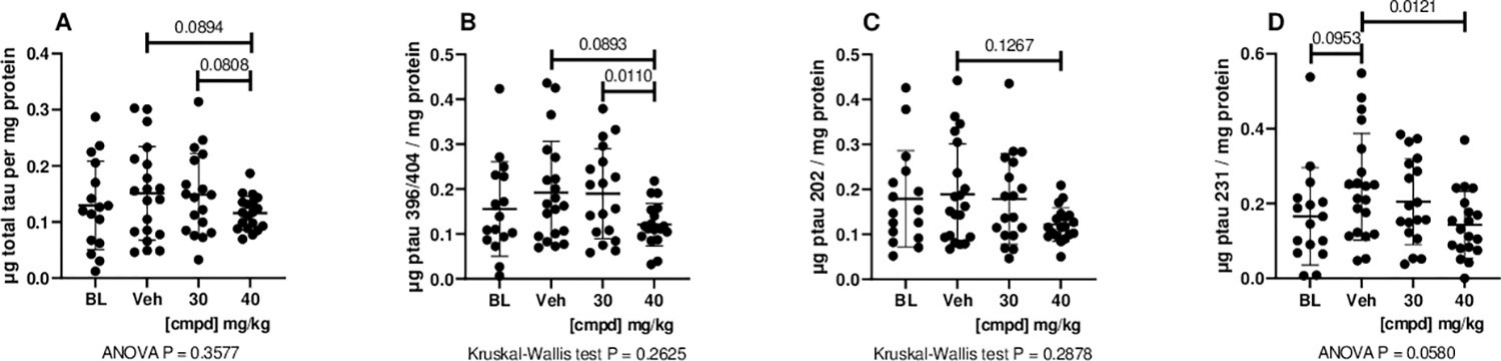
Biochemical analyses of total tau and ptau in Sarkosyl-insoluble preparations from the hindbrain. Tau sandwich ELISAs for total tau and ptau epitopes were performed with capture antibodies (A) DA31, (B) PHF1, (C) CP13, and (D) RZ3 and all ELISAs used reporter antibody DA9-HRP as described (Forest et al., 2013). 30, 30 mg/kg dose group; 40, 40 mg/kg dose group; BL, Baseline; cmpd, compound; ptau, phosphorylated tau; Veh, Vehicle.

Quantitative ELISAs were used to evaluate the Sarkosyl-insoluble fractions from the hindbrain and cortex for total tau and tau phosphorylated at 3 different epitopes. The increase in signal from the Baseline group (3 months) to Vehicle group (7 months) was prevented by treatment with 40 mg/kg (Fig 2). The 40 mg/kg dose group had statistically significantly reduced levels of tau phosphorylated at Ser396/404 compared with the Vehicle group (Fig 2B) and had values that were less scattered than in the other groups.

### Treatment reduced phosphorylation of Sarkosyl-insoluble tau in the hindbrain

**Fig 3:**
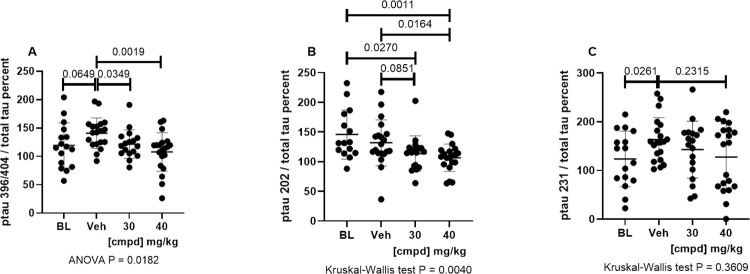
Insoluble ptau normalized to total Sarkosyl-insoluble tau in the hindbrain. Values for ptau were normalized to total tau in the insoluble fraction and presented as percent insoluble ptau normalized to total insoluble tau. (A) mAb PHF1 (ptau396/404) / mAb DA31 (total tau); (B) mAb CP13 ptau202 / mAb DA31 (total tau); (C) mAb RZ3 (ptau231) / DA31 (total tau). 30, 30 mg/kg dose group; 40, 40 mg/kg dose group; BL, Baseline; cmpd, compound; ptau, phosphorylated tau; Veh, Vehicle.

Normalizing the ptau values to total tau in the Sarkosyl-insoluble fraction similarly showed a dose-dependent reduction of insoluble tau phosphorylated at the 3 epitopes, indicating that phosphorylation was reduced in addition to reduction of insoluble tau (Fig 3).

There was a statistically significant reduction of insoluble ptau 396/404 in the 30 and 40 mg/kg dose groups compared with the Vehicle group (Fig 3A). Levels of ptau 202 were reduced below the Baseline level, with statistical significance in the 40 mg/kg dose group (Fig 3B) compared with the Vehicle group. Levels of ptau 231 also trended lower with dose dependence (Fig 3C).

### Treatment increased proteolysis of Sarkosyl-insoluble tau in the hindbrain

**Fig 4:**
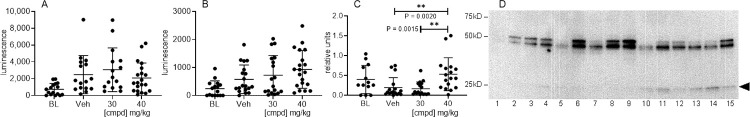
Analysis of Sarkosyl-insoluble tau from the hindbrain by PHF1 immunoblot. Quantification of immunoblot signal of human and mouse tau doublet at (A) ~50 kDa and (B) ~25 kDa fragment. (C) The ratio of ~25 kDa fragment to ~50 kDa bands. Panel D is a representative blot showing Baseline (lanes 1–4); Vehicle (lanes 5–8), 30 mg/kg dose group (lanes 9–11), and 40 mg/kg dose group (lanes 12–15). The arrow in panel D indicates the 25 kDa tau fragment containing the ptau 396/404 epitope. The same preparations that were evaluated by ELISA in Fig 1 were resolved by 4–20% SDS-PAGE (Criterion TGX Gel, Bio-Rad Laboratories, Inc., Hercules, CA) run with reducing agent and transferred to a PVDF membrane (Trans-Blot Turbo Transfer System, Bio-Rad). The FluorChem R System (ProteinSimple, San Jose, CA) was used to capture images, and quantification of chemiluminescent signal was performed using AlphaView software (ProteinSimple). 30, 30 mg/kg dose group; 40, 40 mg/kg dose group; BL, Baseline; Veh, Vehicle.

Immunoblot analyses of Sarkosyl-insoluble fractions from the hindbrain were performed with mAb PHF1 (Fig 4). Size resolution of the ptau species showed 2 predominant bands. The 50 kDa band is consistent with the apparent molecular weight of the human P301L tau 4R0N construct expressed in JNPL3 mice, and the band above may be a larger endogenous murine tau isoform phosphorylated at Ser 396/404 (Fig 4D). The accumulation of a tau fragment with an apparent molecular weight of 25 kDa is increased with dose (Fig 4B and 4D, lanes 9–14). The ratio of the level of fragment to the levels of higher molecular weight bands for the 40 mg/kg dose group is significantly higher than for the Vehicle group or 30 mg/kg dose group (Fig 3C), suggesting increased proteolytic processing with treatment.

**Fig 5:**
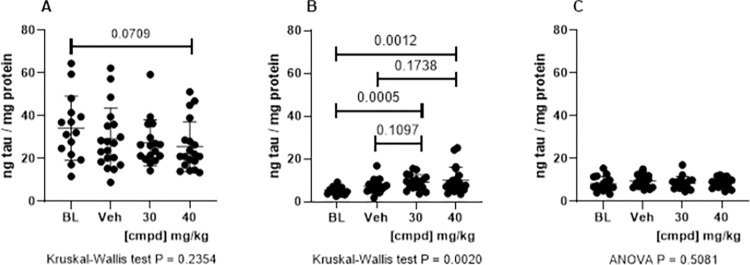
Self-associated tau. Mono-antibody ELISAs formatted with mAb DA9 for capture and DA9-HRP for reporter were used to measure self-associated tau. (A) hindbrain, (B) cortex, (C) hippocampus. 30, 30 mg/kg dose group; 40, 40 mg/kg dose group; BL, Baseline; cmpd, compound; Veh, Vehicle.

The primary endpoint for this study was reduction of Sarkosyl-insoluble tau with statistical significance, signaling the compound’s efficacy for inhibiting tau aggregation. However, the approach to select the inhibitors was based on in vitro assays of tau self-association inhibition. Therefore, to determine whether the compound produced a similar effect in vivo, levels of self-associated tau were determined by ELISA using a mono-antibody format in which both the capture and reporter antibodies are the same and bind to a single epitope in tau. Tau monomers do not produce signal, as the capture antibody binding the epitope blocks binding of the reporter antibody. Self-associated tau, on the other hand, has at least one more epitope to which the reporter antibody can bind and produce signal. Self-associated tau was measured in homogenates prepared without denaturing conditions from the hindbrain, hippocampus, or cortex (Fig 5). In the hindbrain, there was a decrease in self-associated tau in the Vehicle and treatment groups compared with Baseline, but self-associated tau decreased in the treatment groups relative to the Vehicle group (Fig 5A). No statistically significant differences in levels of self-associated tau were found between the Vehicle and treatment groups in the cortex and hippocampus (Fig 5B and 5C).

### Treatment caused trend reduction of total tau in the hindbrain

**Fig 6:**
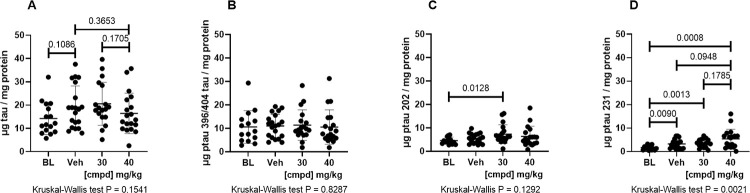
Total tau and ptau in HS preparations from the hindbrain. Tau sandwich ELISAs for total tau and ptau epitopes were performed with capture antibodies (A) DA31, (B) PHF1, (C) CP13, and (D) RZ3; all ELISAs used reporter antibody DA9-HRP as described (Forest et al., 2013). 30, 30 mg/kg dose group; 40, 40 mg/kg dose group; BL, Baseline; cmpd, compound; ptau, phosphorylated tau; Veh, Vehicle.

Heat-stable preparations of tau were used to evaluate the overall levels of tau in the hindbrain, cortex, and hippocampus. Heat treatment dissociates tau from noncovalent interactions with other proteins that also precipitate upon denaturing, with heat enabling the purification of HS tau protein. No significant differences in total tau were observed among groups (Fig 6). However, there was a trend increase in the level of total tau in the Vehicle group compared with the Baseline group and the 40 mg/kg dose group (Fig 6A).

### There is a negative correlation between ptau 231 in heat stable and Sarkosyl-insoluble fractions in the hindbrain in the high-dose group

**Fig 7:**
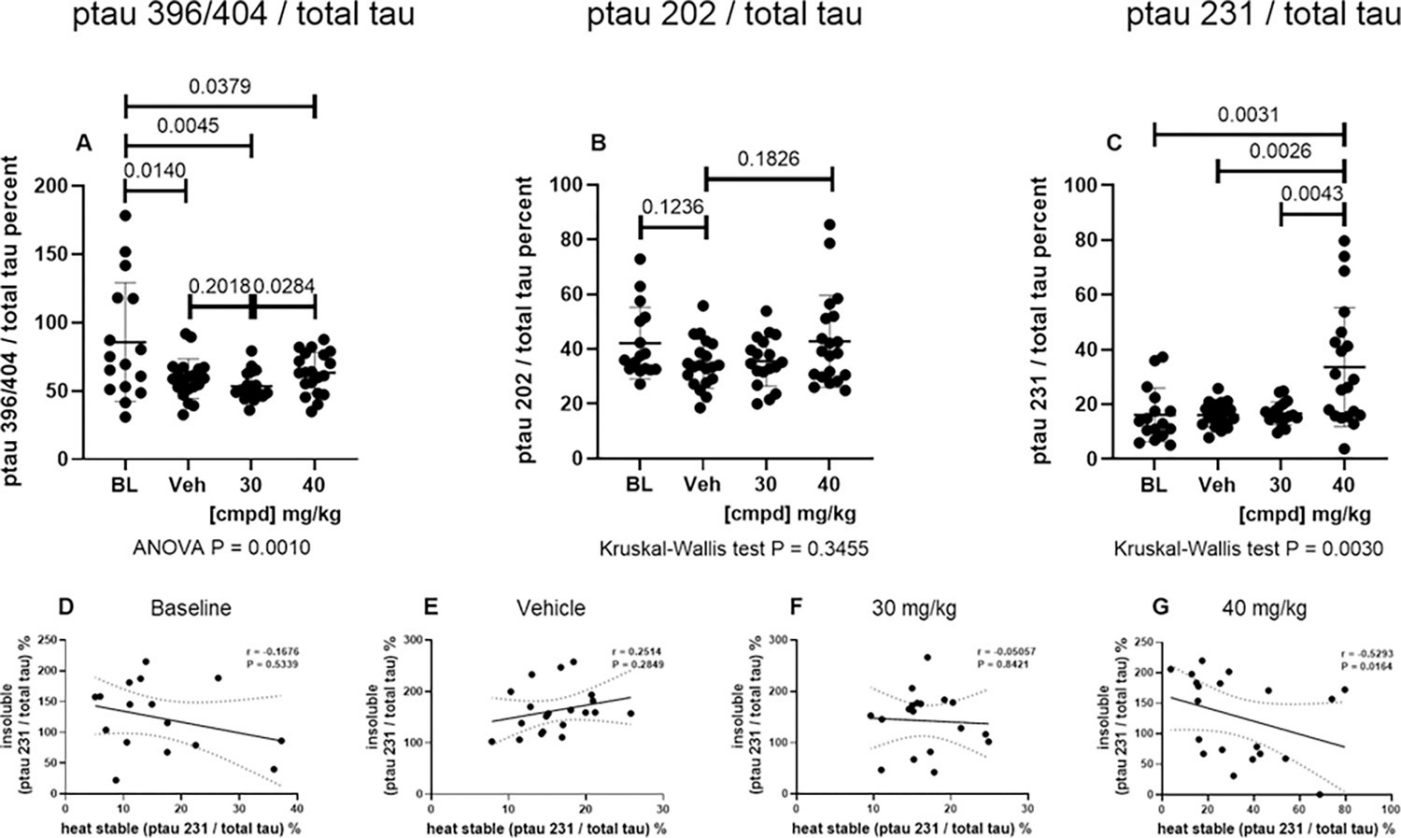
ptau normalized to total tau in HS preparations from the hindbrain. The levels of ptau were normalized to total tau for (A) ptau 396/404, (B) ptau 202, and (C) ptau 231 from ELISAs of HS preparations from the hindbrain. The normalized values for ptau 231 in the Sarkosyl-insoluble fraction were plotted against the values in the HS fraction from the hindbrain for the (D) Baseline group, (E) Vehicle group, (F) 30 mg/kg dose group, and (G) 40 mg/kg dose group.30, 30 mg/kg dose group; 40, 40 mg/kg dose group; BL, Baseline; cmpd, compound; ptau, phosphorylated tau; Veh, Vehicle.

To evaluate tau phosphorylation in the HS fraction independent of total tau levels, the values for ptau were normalized to total tau (Fig 7). The Baseline group had significantly more ptau 396/404 than the Vehicle and Treatment groups (Fig 7A), and the 40 mg/kg dose group had a significantly greater level of ptau 231 (Fig 7C). The higher levels in the Baseline and high dose groups were driven in part by normalization to the lower levels of total tau in these groups (Fig 6A). Correlations between ptau 231 in the HS and Sarkosyl-insoluble fractions from the hindbrain were plotted in individual mice to evaluate the relationship between these values (Fig 7D–7G). The Vehicle group had a weak positive correlation (Fig 7E), the Baseline group and 30 mg/kg dose group did not have significant correlations (Fig 7D and 7F), and the 40 mg/kg dose group had a statistically significant negative correlation (Fig 7G).

**Fig 8:**
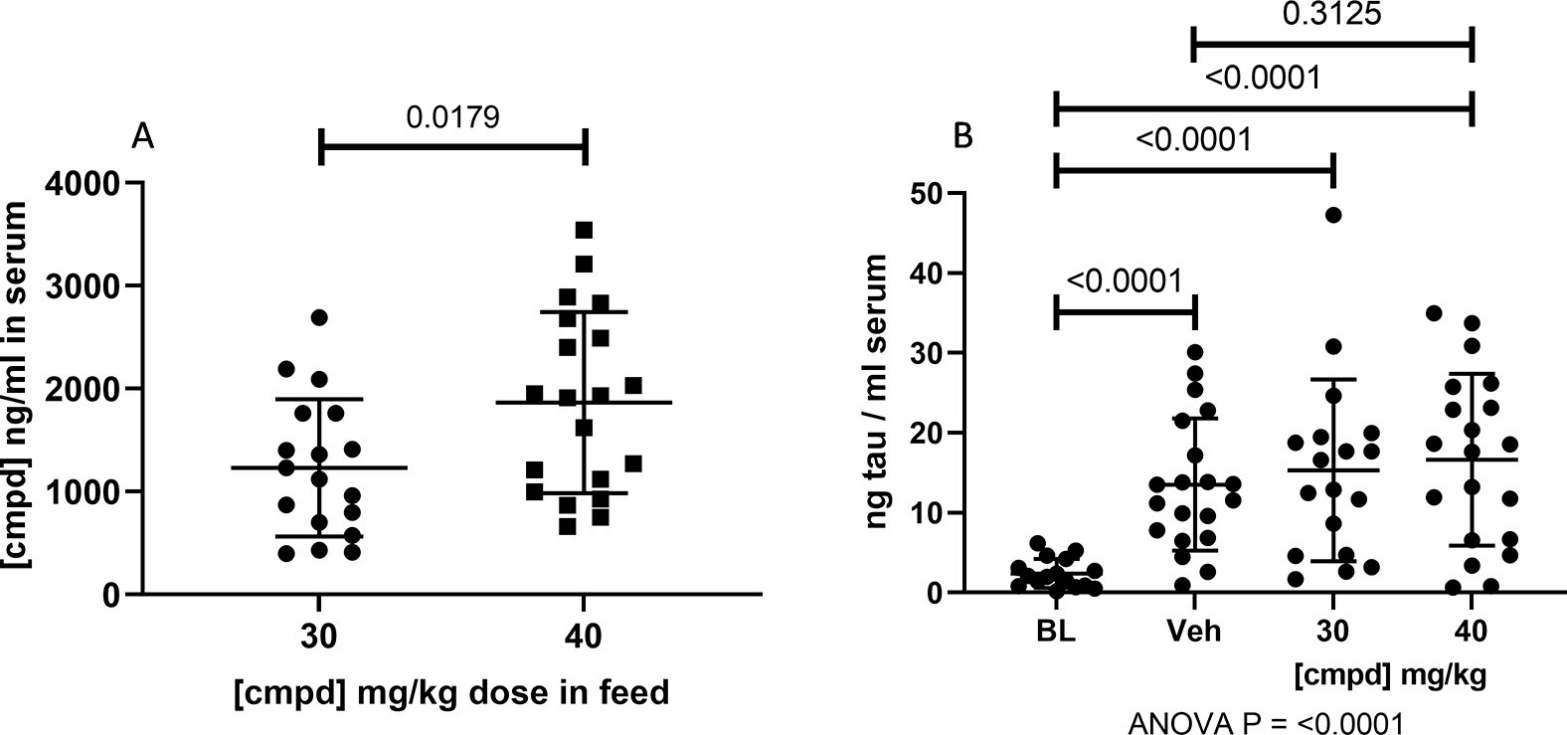
Serum compound levels and total tau. (A) LC/MS/MS was used to determine compound levels in the serum from the treatment groups. (B) Heat treatment of serum samples at acidic pH was used to remove interfering antibodies from the samples to enable quantification of tau by ELISA (d’Abramo et al., 2013). 30, 30 mg/kg dose group; 40, 40 mg/kg dose group; BL, Baseline; cmpd, compound; Veh, Vehicle.

Serum samples from the mice were used to determine compound exposure and levels of tau. The compound exposure in the serum was determined using LC/MS/MS (Fig 8A). The compound level increased proportionally with the dose (administered in the feed), and this increase contributed to the spread in data at the study endpoint. Serum samples were also analyzed by ELISA to ascertain levels of total tau. There was a statistically significant increase in serum tau from the Baseline to Vehicle and Treatment groups, but there was no statistically significant difference between the Vehicle and Treatment groups (Fig 8B).

## Discussion and conclusions

This study evaluated the effect of preventive treatment of young P301L tau JNPL3 mice from 3 to 7 months of age with an orally available small molecule targeting tau self-association. Biochemical analysis of the groups showed that the 40 mg/kg dose inhibited the accumulation of Sarkosyl-insoluble tau in the hindbrain above baseline, as well as its phosphorylation at 3 distinct epitopes. Immunoblot analyses of these samples showed increased levels of a ptau 396/404 fragment in the 40 mg/kg dose group, which may suggest that treatment led to greater proteolytic processing of tau.

The correlation between insoluble ptau 231 and HS ptau 231 in the hindbrain was dependent on treatment. There was a positive correlation in the Vehicle group and a negative correlation in the 40 mg/kg dose group, perhaps suggesting that treatment may prevent the aggregation of ptau 231.

It was anticipated that these mice would develop a more advanced phenotype at a young age than htau mice that express nonmutated human tau and would provide a greater dynamic range to evaluate the effect of the compound. There were no adverse effects related to treatment in the study, and the compound exposure was proportional to dose as determined by analysis of the serum samples at the study endpoint. Analyses of tau in Sarkosyl-insoluble preparations were performed for the hindbrain and cortex, and analyses were performed on HS fractions from all three regions. The Vehicle group had higher levels of insoluble tau than the Baseline group in the hindbrain, but the levels were similar in the cortex, indicating that the pathological phenotype of these mice was beginning to emerge at the endpoint of the study and was delayed compared to the model’s original characterization [34]. The results of the preventive treatment studies in htau and JNPL3 mice support the evaluation the activity of OLX-07010 in the context of a more advanced phenotype in the Vehicle group. A similar preventive study could be performed with an extended treatment window, to 12 months of age, at which time there should be extensive tau aggregates in the cortex and the emergence of the hindlimb motor impairment phenotype. A therapeutic paradigm could be used to determine the effect of the molecule with treatment from 7 to 12 months. A mouse model expressing human P301S tau, such as PS19 [22, 37] that has been shown to have a more aggressive timeline for developing a tauopathy phenotype, could also enable a more informative preventive study. However, to evaluate the potential of this compound for treating AD, a transgenic model developing both tau and beta-amyloid aggregates is necessary. In such a model, the accumulation of beta-amyloid may lead to inflammation and tau hyperphosphorylation, contributing to tau loss of function and aggregation [38, 39].

Small molecule drugs have advantages over immunotherapeutic approaches targeting tau [2, 40–44]. In brief, orally available small molecules can be designed to penetrate the blood–brain barrier, to enter cells where tau is aggregating, and to access extracellular vesicles that may be vectors of tau seeds that spread tau pathology [45]. Meanwhile, only about 0.1% of intravenously administered antibodies are found in the cerebrospinal fluid [46]. Our program discovers small molecules that are agnostic to specific posttranslational modifications, conformational changes, or strains of tau aggregates and are a low-cost therapeutic intervention or preventive therapy relative to passive immunotherapy.

## Supporting information

S1 Raw image (TIF)
