# Supplementary figures and images for: Small molecule inhibitor of tau self-association in a mouse model of tauopathy: A preventive study in P301L tau JNPL3 mice
Source: PLoS One. 2023 Aug 9;18(8):e0286523. doi: 10.1371/journal.pone.0286523 (PMC10411817; doi:10.1371/journal.pone.0286523)

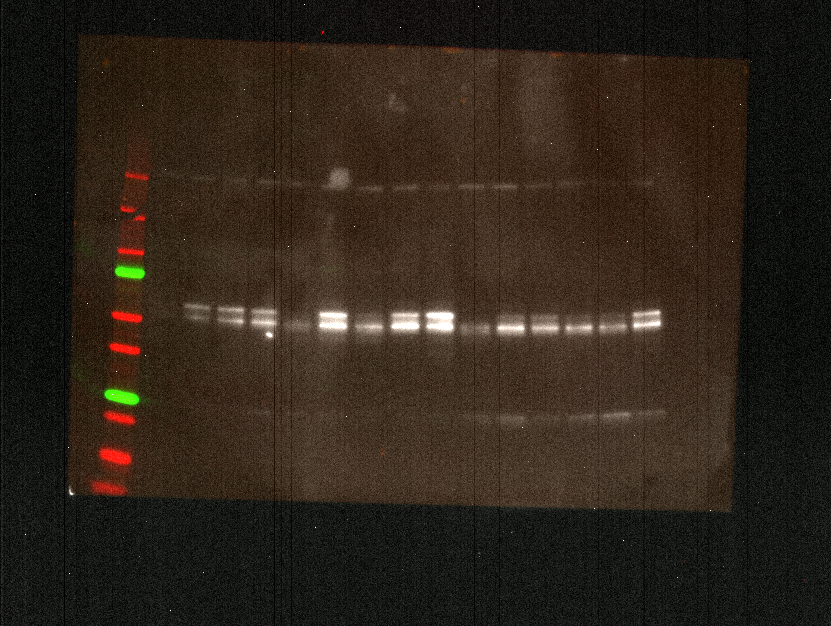

Supplement: S1 Raw image — (TIF) [file pone.0286523.s001.tif]
